# A meta-analysis of ventriculostomy-associated cerebrospinal fluid infections

**DOI:** 10.1186/s12879-014-0712-z

**Published:** 2015-01-08

**Authors:** Mahesh Ramanan, Jeffrey Lipman, Andrew Shorr, Aparna Shankar

**Affiliations:** Burns Trauma Critical Care Research Centre, School of Medicine, University of Queensland, Brisbane, Queensland Australia; Department of Intensive Care Medicine, Royal Brisbane and Women’s Hospital, Herston, Brisbane, Queensland 4029 Australia; Medical Intensive Care Unit, Washington Hospital Center, Washington, DC USA; Kempegowda Institute of Medical Sciences, Bangalore, Karnataka India

**Keywords:** Cerebral ventriculitis, Cerebrospinal fluid, Ventriculostomy, Catheter-related infection, Neurosurgery, Meta-analysis

## Abstract

**Background:**

Ventriculostomy insertion is a common neurosurgical intervention and can be complicated by ventriculostomy-associated cerebrospinal fluid infection (VAI) which is associated with increased morbidity and mortality. This meta-analysis was aimed at determining the pooled incidence rate (number per 1000 catheter-days) of VAI.

**Methods:**

Relevant studies were identified from MEDLINE and EMBASE and from reference searching of included studies and recent review articles on relevant topics. The Newcastle-Ottawa Scale was used to assess quality and risk of bias. A random effects model was used to pool individual study estimates and 95% confidence intervals (CI) were calculated using the exact Poisson method. Heterogeneity was assessed using the heterogeneity χ2 and I-squared tests. Subgroup analyses were performed and a funnel plot constructed to assess publication bias.

**Results:**

There were a total of 35 studies which yielded 752 infections from 66,706 catheter-days of observation. The overall pooled incidence rate of VAI was 11.4 per 1000 catheter days (95% CI 9.3 to 13.5), for high quality studies the rate was 10.6 (95% CI 8.3 to 13) and 13.5 (95% CI 8.9 to 18.1) for low quality studies. Studies which had mean duration of EVD treatment of less than 7 days had a pooled VAI rate of 19.6 per 1000 catheter-days, those with mean duration of 7–10 days had VAI rate of 12.8 per 1000 catheter-days and those with mean duration greater than 10 days had VAI rate of 8 per 1000 catheter-days. There was significant heterogeneity for the primary outcome (p = 0.004, I-squared = 44%) and most subgroups. The funnel plot did not show evidence for publication bias.

**Conclusions:**

The incidence rate of VAI is 11.4 per 1000 catheter-days. Further research should focus on analysis of risk factors for VAI and techniques for reducing the rate of VAI.

## Background

External ventricular drains (EVD) or ventriculostomies are commonly used in neurosurgical patients to monitor and treat raised intracranial pressure, drain intraventricular blood and temporarily treat acute hydrocephalus. Ventriculostomies are associated with a range of potential infectious and non-infectious complications [1]. Whilst rare infectious complications such as skull osteomyelitis, subdural empyema, brain abscess and distant infection are possible, the most common and clinically significant infectious complication is ventriculostomy-associated cerebrospinal fluid (CSF) infection (VAI). The VAI rate in the literature is variable, with individual studies reporting rates from 1% to 45% [2-36]. Reviews using non-meta-analytic techniques have found that 8.8% and 9.5% of patients with ventriculostomies develop VAI. VAI is associated with increased morbidity and mortality, longer intensive care unit and hospital stay, and increased healthcare costs [2,13,31].

Furthermore, there are various clinical dilemmas faced by physicians caring for patients with ventriculostomies with regard to prompt diagnosis, investigation and treatment of VAI. Clinical signs such as fever, altered consciousness, nuchal rigidity, emesis and focal neurological deficits are severely confounded by the primary neurological insult, treatments directed at preventing secondary neurological injury (sedation, neuromuscular blockade), seizures, electrolyte disturbances and non-neurological infections. CSF signs are confounded by intraventricular or subarachnoid blood, neurosurgical interventions, systemic antimicrobial therapy and antibiotic-impregnated catheters (AIC). Ventriculostomies, like other devices inserted through the skin, can become colonized by skin organisms. Colonization is not necessarily indicative of CSF infection, though CSF cultures may be positive. These difficulties are reflected in the lack of consensus definition for VAI, uncertainty regarding the trigger for empirical antibiotic therapy and the resultant wide variations in practice relating to both.

The primary aim of this systematic review and meta-analysis was to determine the pooled incidence rate of VAI. Secondary aims were to explore factors (such as duration of ventriculostomy treatment, age group, CSF infection definition, CSF culture frequency) associated with the incidence rate of VAI and to describe the microbiological findings associated with VAI. This study was conducted using the PRISMA guidelines for conduct of systematic reviews [37].

## Methods

### Selection criteria

All observational studies, both retrospective and prospective, that reported VAI rate were included. Randomized controlled trials of interventions were not included. When duplicate cohorts were identified, the results from the most recent publication were included to avoid overlap.

Patients with ventriculostomies were included, regardless of age or underlying diagnosis. Patients with intracranial pressure monitors or internalized CSF shunts were excluded.

Studies that reported VAI rates as infections per 1000 catheter-days and studies where this could be calculated from published data were included. We anticipated that various different definitions of VAI would be identified and all of these definitions were included.

### Search methods

Relevant studies of all languages were identified from MEDLINE (1966–2013) and EMBASE (1966–2013) databases (search strategy in Table 1). The references of all included studies and relevant reviews [1,38-41] were searched for additional studies. The literature and reference search were performed by M.R. in June 2013.

**Table 1:** **MEDLINE and EMBASE search strategy**

1.	exp Ventriculostomy/
2.	(external adj25 ventricular).tw.
3.	(ventricular drain or ventricular catheter).tw.
4.	exp Cerebrospinal Fluid Shunts/ or exp Ventriculoperitoneal Shunt/
5.	or/1-4
6.	exp Postoperative Complications/ or exp Surgical Wound Infection/ or exp Bacterial Infections/
7.	exp Antibiotic Prophylaxis/
8.	exp Cerebral Ventriculitis/ or exp Meningitis/
9.	ventriculitis.tw.
10.	(cerebrospinal adj25 infection).tw.
11.	or/6-10
12.	5 and 10

Titles and abstracts were reviewed, and relevant studies were selected for full text review. Studies which met the pre-specified inclusion criteria on full text review were selected for inclusion in the meta-analysis. Reasons for exclusion were recorded. The full text review was performed by two authors (M.R. and Ap.S.) with disputes resolved by discussion.

### Data collection

A data collection spreadsheet was created in Microsoft Excel 2010 and data extracted by two authors (M.R. and Ap.S.). The following data were extracted from each selected study; publication year, country, study type (retrospective or prospective), gender, mean age, numbers of patients, ventriculostomies, catheter-days and VAI’s. The following data were extracted for subgroup analyses and secondary objectives; CSF culture frequency, VAI definition, underlying diagnosis, antibiotics, AIC, duration of ventriculostomy and microbiological findings.

### Quality appraisal

Quality appraisal was conducted using the Newcastle-Ottawa scale (NOS) [42]. For cohort studies, the NOS is scored out of nine stars, four for selection, two for comparability and three for outcome. For this review, four of the nine items were relevant (“representativeness of the exposed cohort”, “demonstration that outcome of interest not present at start of study”, “assessment of outcome” and “adequacy of follow-up”). We defined studies that scored zero to two stars as low quality, and studies that scored three or four stars as high quality for sensitivity analysis purposes.

### Statistical analysis

The outcome of interest was incidence rate of VAI per 1000 catheter-days. 95% confidence intervals (CI) were calculated for individual studies using an exact Poisson method in Statistical Analysis Software 9.2 (SAS, Cary, NC, USA) with PROC GENMOD. The meta-analysis was performed in Microsoft Excel 2010 [43] using the random effects method [44] to determine the pooled incidence rate. Forest plots were created to provide visual representation of the data and were inspected for heterogeneity. For objective measures of heterogeneity, the heterogeneity χ2 and I-squared tests [45] were performed. A significance threshold of p = 0.05 was applied to the heterogeneity χ2. I-squared values less than 25% were defined as low heterogeneity, 25-50% as moderate and greater than 50% as high heterogeneity.

Pre-specified subgroup analyses were performed to test the effect of study type (retrospective versus prospective), AIC usage, infection definition, culture frequency, publication year, age group and duration of ventriculostomy insertion on the incidence rate of VAI. Sensitivity analysis was performed by examining the effect of removing small studies (defined as 1000 catheter-days or less) and low quality studies (defined as one or two stars on the Newcastle-Ottawa scale). A funnel plot [46] was examined for evidence of publication bias.

## Results

### Literature search

**Figure 1:**
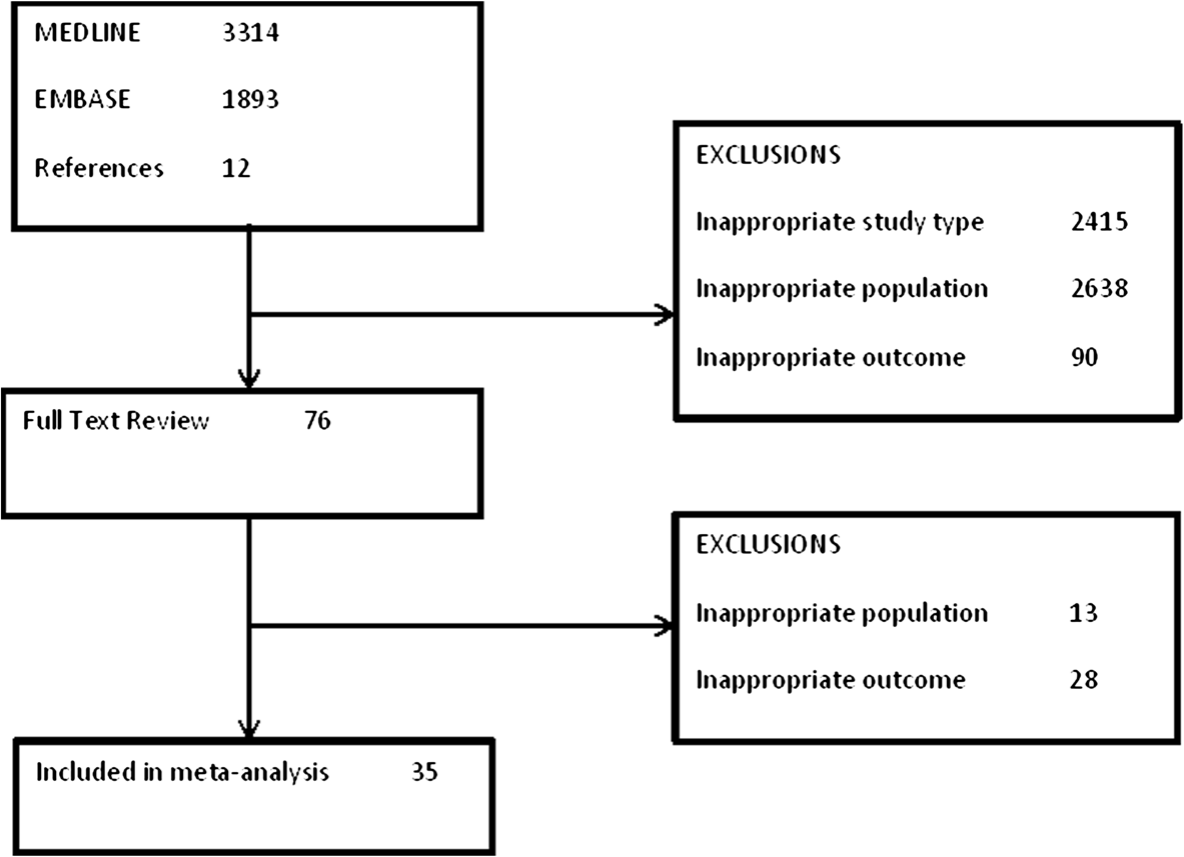
**Flow diagram of the search strategy and exclusions at each stage.**

5219 studies were identified in our search (Figure 1). 3314 were in MEDLINE, 1893 in EMBASE and 12 in the reference search. Out of these, 76 were chosen for full text review from which 35 met pre-specified inclusion criteria and were included in the meta-analysis.

### Study characteristics

The 35 included studies (Table 2) yielded 752 VAI’s from 66,707 catheter-days. There were 6681 patients from 33 studies in which this information was available. Different definitions of VAI were used across the selected studies. The two most common definitions were positive culture in 18, the Centre for Disease Control (CDC) definition [47] in five studies. Of the remaining 12 studies, five used positive culture plus clinical or other microbiological criteria and seven used positive culture or clinical or microbiological criteria to define VAI. AIC were used in four studies (33% to 100% of patients) and were not used in 18 with AIC information unavailable in the remaining 13. Patient characteristics from individual studies are presented in Table 2.

**Table 2:** **Characteristics of included studies**

**Study**	**Year**	**Country**	**Study Type**	**n (patients)**	**n (evd’s)**	**Length of treatment (mean no. days)**	**Age (mean)**	**Males (%)**	**Trauma (%)**	**Cerebrovascular (%)**	**Other indications (%)**	**Antibiotic-impregnated catheters (%)**	**Periprocedural** ^**a**^ **antibiotics (%)**	**Prophylactic** ^**b**^ **antibiotics (%)**	**Culture frequency**	**Infection definition**
Alleyne [31]	2000	USA	Retrospective	308	-	9.3	46.6	50.6	27.9	50.3	21.8	0	100	67.9	twice weekly	culture
Arabi [36]	2005	Saudi Arabia	Retrospective	84	99	7	34	78.6	63.1	21.4	15.5	-	59.6	-	when indicated	culture or wcc^c^ + glucose + clinical signs
Bota [35]	2005	Belgium	Retrospective	638	-	9.5	41.3	56.9	23	62.2	14.7	0	100	-	daily	culture + csf^d^ + clinical
Camacho [8]	2011	Brazil	Prospective	119	130	7.1	44.4	48.7	26.9	53.8	19.3	0	73.1	-	when indicated	unclear
Chi [24]	2009	Taiwan	Retrospective	155	197	18.8	58.1	63.2	-	-	-	-	100	-	when indicated	culture
Dasic [10]	2006	UK	Retrospective	95	113	10.7	54.7	62.1	-	-	-	0	-	-	unclear	culture
Fichtner [28]	2010	Germany	Retrospective	164	-	10	53.5	50	-	56.1	-	0	67.7	0	thrice weekly	culture
Hader [17]	2000	Canada	Retrospective	157	160	5.6	8	0	40.8	7.6	51.6	0	100	43	daily	culture + gram stain or multiple cultures
Hoefnagel [2]	2008	Netherlands	Retrospective	228	-	8.1	58	49.6	3.5	78.1	18.4	0	35.5	-	thrice weekly	culture
Holloway [25]	1996	USA	Retrospective	584	866	7.5	29.9	77.2	100	0	0	-	-	-	when indicated	culture or wcc or csf glucose
Khalil [9]	2005	UK	Retrospective	.	24	12.3	-	-	-	-	-	-	-	-	unclear	culture
Kim [34]	2012	USA	Retrospective	343	-	12.4	53.7	42	-	71	-	0	100	0	when indicated	culture
Kitchen [6]	2011	UK	Retrospective	133	195	7.1	55	35.3	-	-	-	0	-	-	unclear	culture
Lemcke [20]	2012	Germany	Retrospective	95	95	13.7	53.6	53.7	10.5	83.2	6.3	32.6	100	0	tenth daily	culture
Leverstein Van-Dall [21]	2010	Netherlands	Retrospective + Prospective	.	363	9.2	-	-	-	-	-	-	-	-	unclear	cdc definition
Lo [18]	2007	Australia	Retrospective	199	269	8.2	41	64	74	26	0	0	-	-	thrice weekly	cdc definition
Lundberg [16]	2000	Sweden	Prospective	157	157	7.4	-	-	24.8	51	24.2	0	-	-	when indicated or at removal	culture
Lwin [27]	2012	Singapore	Retrospective + Prospective	234	-	7.8	-	65	-	-	-	0	-	-	when indicated	culture
Lyke [13]	2001	USA	Retrospective	157	196	5.3	54.5	42.7	0	0	100		100	100	when indicated	culture + wcc or glucose for low virulence organisms
Mahe [7]	1995	France	Retrospective	53	64	14.6	47.3	-	-	-	-	-	-	-	daily	culture or wcc
McLaughlin [19]	2012	USA	Retrospective	75	97	13.7	59	34.7	0	100	0	100	-	-	when indicated	cdc definition
Moon [12]	2007	Korea	Retrospective	112	174	8.3	48.5	63.4	63.4	26.8	9.8	-	100	100	daily	culture + fever
Park [29]	2004	USA	Retrospective	595	770	8.6	51.3	51.3	12.6	66.7	20.7	-	100	100	when indicated	culture
Rafiq [15]	2011	Pakistan	Retrospective	76	-	11.4	37.9	53.9	-	-	-	0	100	100	when indicated	culture
Rivero-Garvia [26]	2011	Spain	Retrospective	534	648	10.2	-	-	-	-	-	42.8	-	-	when indicated	unclear
Roitberg [30]	2001	USA	Retrospective	103	-	10.7	-	39.8	2.9	85.4	11.7	-	-	-	daily	clinical + wcc
Scheithauer [23]	2009	Germany	Prospective	225	-	11.2	-	-	-	-	-	0	0	0	when indicated	cdc definition
Scheithauer [22]	2010	Germany	Prospective	158	-	14.3	-	-	-	-	-	0	0	0	thrice weekly	cdc definition
Schodel [32]	2012	Germany	Retrospective	166	-	17.1	58.7	44	9	86.1	4.8	0	100	0	unclear	culture + clinical signs
Schultz [11]	1993	USA	Prospective	78	94	11.9	48.8	53.8	37.2	-	-	0	-	94.9	unclear	culture
Sloffer [33]	2005	USA	Retrospective	100	113	11.4	55.6	39	8	72	20	100	100	-	when indicated	culture
Smigoc [5]	2012	Slovenia	Retrospective	48	-	9.8	-	-	-	27.1	-	-	-	-	unclear	culture or clinical signs
Smith [4]	1976	USA	Retrospective	56	65	4	-	-	-	-	-	0	95.4	95.4	unclear	culture
Williams [14]	2011	Australia	Retrospective + Prospective	382	-	5.4	46.1	60.2	32.2	38	29.8		-	-	daily retro, third daily pros	culture
Wyler [3]	1972	USA	Retrospective	70	102	5	7.2	-	0	4.3	95.7	0	-	62.9	insertion, withdrawal and when indicated	culture

^a^Periprocedural = antibiotic administered immediately prior to procedure.

^b^Prophylactic = antibiotic administered for 24 hours or more after the procedure.

^c^wcc = white cell count.

^d^csf = cerebrospinal fluid.

### Quality appraisal

The quality of selected studies was assessed using four items from the NOS (Table 3). 11 studies met all criteria and scored four “stars” whilst 13 scored three, eight studies scored two and three scored one star. No studies scored zero stars. 31 studies scored a star for adequacy of follow-up, 26 for representativeness of the cohort, 24 for assessment of outcome and 21 for demonstration that VAI was not present at the outset.

**Table 3:** **Quality appraisal of included studies using Newcastle-Ottawa Scale**

**Study (Author-Date)**	**Representativeness**	**Demonstration that outcome not present at outset**	**Assessment of outcome (proven infection vs suspected infection based on other criteria)**	**Adequacy of follow-up (<5% loss)**
Alleyne 2000 [31]	*	*	*	*
Arabi 2005 [36]	*			*
Bota 2005 [35]	*	*	*	*
Camacho 2011 [8]	*	*		*
Chi 2009 [24]	*	*	*	*
Dasic 2006 [10]	*		*	*
Fichtner 2010 [28]		*	*	
Hader 2000 [17]	*	*	*	*
Hoefnagel 2008 [2]	*	*	*	
Holloway 1996 [25]				*
Khalil 2005 [9]	*	*	*	*
Kim 2012 [34]	*	*	*	*
Kitchen 2011 [6]			*	*
Lemcke 2012 [20]	*	*	*	*
Leverstein Van-Dall 2010 [21]	*			*
Lo 2007 [18]	*	*		*
Lundberg 2000 [16]		*	*	
Lwin 2012 [27]	*		*	*
Lyke 2001 [13]	*	*	*	*
Mahe 1995 [7]	*			*
McLaughlin 2012 [19]				*
Moon 2007 [12]	*		*	*
Park 2004 [29]	*	*	*	*
Rafiq 2011 [15]	*	*	*	*
Rivero-Garvia 2011 [26]	*			*
Roitberg 2001 [30]				*
Scheithauer 2009 [23]	*	*		*
Scheithauer 2010 [22]	*	*		*
Schodel 2012 [32]	*		*	*
Schultz 1993 [11]		*	*	*
Sloffer 2005 [33]		*	*	*
Smigoc 2012 [5]		*	*	*
Smith 1976 [4]	*		*	
Williams 2011 [14]	*	*	*	*
Wyler 1972 [3]	*		*	*

*Denotes that the quality criteria has been met.

### Main results

#### Incidence

**Figure 2:**
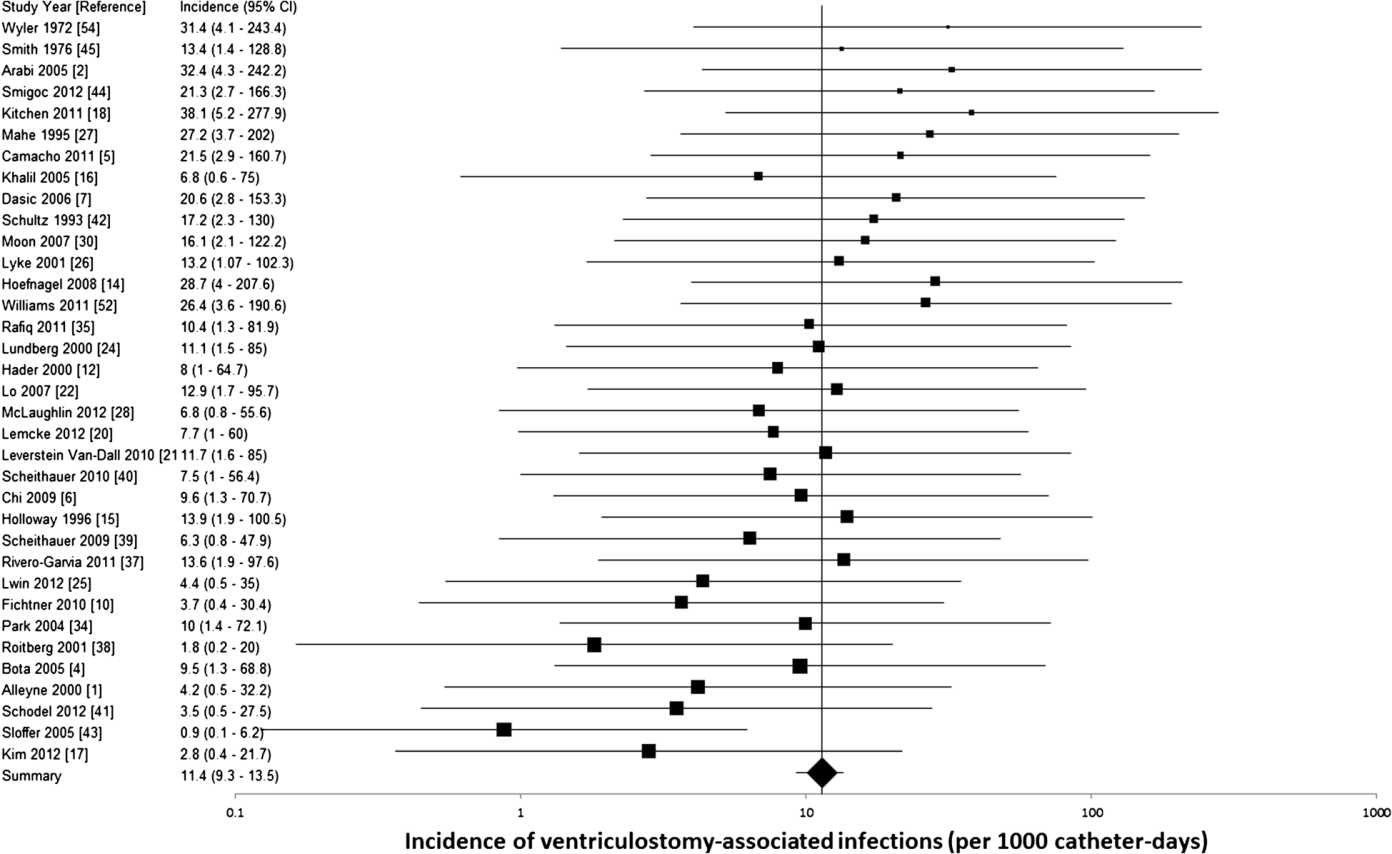
**Forest plot of meta-analysis (random effects model) of ventriculostomy-associated infections. CI confidence interval.**

The 95% CI’s for the individual study estimates are shown on the forest plot (Figure 2). The pooled VAI rate (Figure 2) was 11.4/1000 catheter-days (95% CI 9.3 to 13.5). Significant heterogeneity was detected using the heterogeneity χ2 test (χ2 = 60.19, degrees of freedom (df) = 34, p = 0.004). The I-squared test (I-squared = 45%) was consistent with moderate heterogeneity.

#### Microbiology

**Figure 3:**
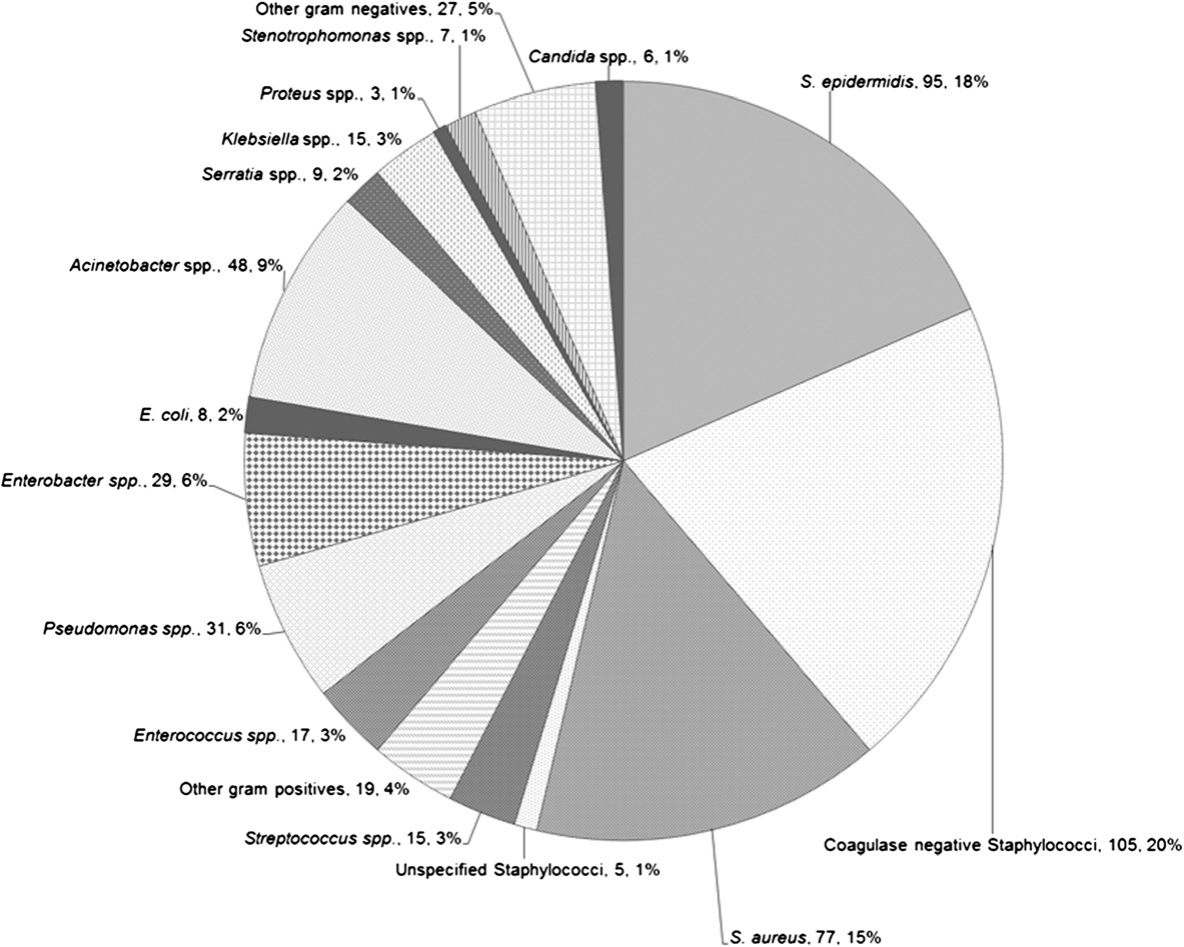
**Pie diagram of breakdown of 523 positive cerebrospinal fluid cultures.**

There were a total of 523 positive cultures from 25 studies which presented microbiological data (Figure 3). 333(64%) of these were gram positive bacteria, 177(35%) gram negative bacteria and 6(1%) were *Candida* spp., 95 were *Staphylococcus epidermidis*, with another 105 reported as coagulase negative staphylococci and 77 *Staphylococcus aureus*(of which 12 were methicillin-resistant). Amongst the gram negative bacteria, *Acinetobacter* spp.(48) were the most common, followed by *Pseudomonas* spp*.*(31) and *Enterobacter* spp.(29). In studies without AIC, 41% (175/425) of positive cultures were gram negative bacteria whereas in studies with AIC, 10% (9/83) were gram negative bacteria.

#### Sensitivity analysis

Sensitivity analyses (Table 4) were performed using NOS and sample size criteria. Including only studies with NOS scores of 3 and 4, the pooled VAI rate was 10.6/1000 catheter-days (95% CI 8.3 to 13) with significant heterogeneity (p = 0.019, I-squared = 41%). The rate was 13.5/1000 catheter-days (95% CI 8.9 to 18.1, heterogeneity p = 0.046, I-squared = 46%) when studies with NOS scores of 1 and 2 only were included. With studies having sample sizes 1000 catheter-days and under, the VAI rate was 18.3/1000 catheter-days (95% CI 13.4 to 23.3) with low, insignificant heterogeneity (p = 0.34, I-squared = 11%). The rate was 9/1000 catheter-days (95% CI 6.8 to 11.2) when studies with sample size greater than 1000 catheter-days were analyzed. Heterogeneity remained significant (p = 0.027, I-squared = 40%).

**Table 4:** **Subgroup and sensitivity analyses**

**Comparison**	**n**	**Infections/1000 catheter-days**	**Lower 95% CI** ^**a**^	**Upper 95% CI**	**p**	**I-squared (%)**
Overall	35	11.4	9.3	13.5	0.004	44
**Subgroups**						
**Culture frequency**						
Daily culture	5	10.8	4.8	16.8	0.16	39
Culture when indicated	15	11.2	8.6	13.9	0.16	27
Other culture regimes	15	13.5	9.6	17.4	0.048	41
**Infection definition**						
Culture proven infection	18	11.5	8.4	14.6	0.004	54
CDC definition of infection	5	8.8	6.3	11.3	0.403	0.50
Culture plus other features	5	9.1	4.8	13.4	0.436	0
Culture or other features	7	17	10	24.1	0.347	11
**Age group**						
Paediatric	3	10.6	6.1	15.2	0.429	0
Adult	32	11.5	9.3	13.7	0.002	48
**Year**						
Publication year <2000	5	18.3	12.4	24.3	0.39	3
Publication year 2000+	30	10.5	8.4	12.6	0.002	47
**Duration**						
Mean duration <7 days	6	19.6	10	24.1	0.6	0
Mean duration 7-10 days	14	12.8	9.5	16	0.03	49
Mean duration >10 days	15	8	5.4	10.5	0.106	37
**Study type**						
Prospective	5	11	6.6	15.4	0.23	29
Retrospective	30	11.4	9.1	13.8	0.003	47
**AIC**						
No AIC	18	10.8	8.1	13.6	0.001	57
Unclear	13	13.7	9.8	17.6	0.19	25
AIC used	4	7.2	2.6	14.1	0.64	0
**Sensitivity Analysis**						
**Study size**						
<=1000 catheter-days	13	18.3	13.4	23.3	0.34	11
>1000 catheter-days	22	9	6.8	11.2	0.027	40
**Newcastle-Ottawa score**						
1 and 2	11	13.5	8.9	18.1	0.046	46
3 and 4	24	10.6	8.3	13	0.019	41

^a^CI = confidence interval.

#### Subgroup analyses

Subgroup analyses (Table 4) were performed based on age group, publication year, infection definition, study type, AIC, duration of ventriculostomy insertion and CSF culture frequency. The studies which defined VAI as positive CSF culture had a pooled VAI rate of 11.5/1000 catheter-days, those which used the CDC definition had a rate of 8.8/1000 catheter-days, those which used positive culture plus other clinical or microbiological features had a rate of 9.1/1000 catheter-days and those which used positive culture or other clinical or microbiological features had a rate of 17/1000 catheter-days. Studies which had mean duration of EVD treatment of less than 7 days had a pooled VAI rate of 19.6/1000 catheter-days, those with mean duration of 7–10 days had VAI rate of 12.8/1000 catheter-days and those with mean duration greater than 10 days had VAI rate of 8/1000 catheter-days. Studies in which AIC were used had a VAI rate of 7.2/1000 catheter-days whilst the remaining studies had a pooled rate of 12.1/1000 catheter-days. Studies published earlier than 2000 had a higher VAI rate of 18.3/1000 catheter-days compared to 10.5/1000 catheter-days for studies published 2000 or thereafter. Prospective and retrospective studies had similar VAI rates of 11 and 11.4/1000 catheter-days respectively.

#### Funnel plot

**Figure 4:**
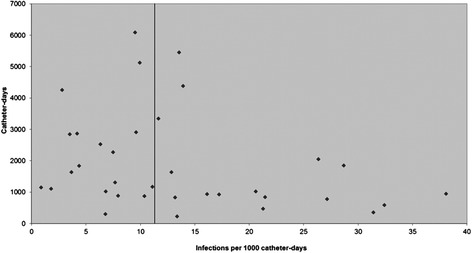
**Funnel plot of incidence of ventriculostomy-associated cerebrospinal fluid infection versus catheter-days.**

A funnel plot was created by plotting catheter-days versus VAI rate (Figure 4) and visually inspected for evidence of publication bias. The funnel plot revealed an excess of smaller studies with extreme estimates, both high and low, of VAI rate. There was a paucity of larger studies with extreme estimates of VAI rate. Both observations were consistent with low likelihood of publication bias.

## Discussion

This meta-analysis of 35 observational studies has calculated a pooled incidence rate of VAI of 11.4/1000 catheter-days (95% CI 9.3-13.5). This translates to 1 VAI per 88 catheter-days. This result is a benchmark against which local practices can be compared. There was, however, significant heterogeneity for the pooled result (p = 0.004, I-squared = 44%) and most subgroup analyses. The reasons for this heterogeneity are numerous and include variations in definition of VAI, antibiotic usage, CSF investigation regimes, patient population, publication year, duration of ventriculostomy insertion, type of catheter, length of subcutaneous tunnel and potentially other unknown factors that cannot be addressed in this meta-analysis.

In comparison to previous studies [34,38] which simply added and averaged percentages to generate an overall estimate of VAI rate, we have performed, to our knowledge, the first systematic review using validated meta-analytic techniques to determine the pooled VAI rate from all published studies. The VAI rate of 11.4/1000 catheter-days can be used by individual institutions as a benchmark for comparison with their local VAI rates. The effect of interventions and changes to local practice can be quantified and compared using this rate. It can be used by future individual studies or meta-analyses of interventions aimed at reducing VAI rate to calculate numbers needed to treat. Further applications would include health economic calculations of the cost of VAI and the quantification of benefits of reducing the VAI rate [48].

From the 25 studies which reported microbiological data from 523 positive cultures, 64% of culture positive infections were caused by gram positive bacteria, predominantly coagulase negative staphylococci (39%, including *S. epidermidis*) and *S. aureus*(15%). A minority of infections (1%) were caused by *Candida* spp., and the rest (35%) by gram negative bacteria including *Acinetobacter* spp.(9.3%) and *Pseudomonas* spp.(6%) followed by enteric organisms. Traditional thinking is that skin flora, predominantly staphylococcal species, which gain entry to the subarachnoid or intraventricular space through the ventriculostomy tract cause VAI. Prophylactic and empirical antibiotics are frequently selected on this basis. Further research is required to differentiate between risk factors for gram positive and gram negative VAI, however, our findings suggest that empirical antibiotic regimes that are not active against gram negative bacteria will be inadequate in about a third of all VAI cases. Furthermore, *Acinetobacter* and *Pseudomonas* species, which are resistant to a broader range of antibiotics than the enteric gram negatives, were the most common gram negative bacteria.

Smaller studies (<1000 catheter-days) reported a much higher incidence rate of VAI (18.3/1000 catheter-days) than larger (>1000 catheter-days) studies (9/1000 catheter-days). This suggests that sampling error may have biased the overall result and the estimate from the larger studies may be more reflective of the true VAI rate. Subgroup analyses revealed similar VAI rates when studies were grouped according to age and culture regime. Some individual studies have suggested that frequent manipulation of ventriculostomies is a risk factor for VAI [2,49]. Our results, however, show very similar rates of VAI (10.8 and 11.2/1000 catheter-days) when comparing studies that performed daily CSF cultures with clinically indicated CSF cultures. An explanation for this is that ventriculostomies are accessed for reasons other than CSF cultures such as flushing the system when blocked with blood clots, other diagnostic tests (biochemical, cytological, immunological) and administering intrathecal medications.

Studies published prior to 2000 had a higher VAI rate (18.3/1000 catheter-days) compared to those published 2000 and thereafter (10.5/1000 catheter-days). This may be a reflection of the evolution and improvement in infection control and prevention practices over time.

The use of AIC for prevention of VAI is controversial. Whilst various analyses [40,41] have suggested a protective effect, the findings presented by Stevens et al. [50] have raised doubt as to whether the incidence of VAI is truly reduced or whether the false negative rate is increased by AIC. Our results show that studies in which AIC were utilized had a pooled VAI rate of 7.2/1000 catheter-days, considerably lower than 12.1/1000 catheter-days in studies in which AIC were not used or AIC usage was unclear. The latter rate is perhaps closer to the true rate of VAI with confounding by AIC usage removed.

There is no universally accepted definition of VAI, as evidenced by the various definitions encountered during this meta-analysis. 18 out of 35 studies defined VAI as micro-organism growth on CSF culture, and the pooled VAI rate amongst these was 11.5/1000 catheter-days. Those that used the CDC definition [47], with or without minor modifications, had a rate of 8.8/1000 catheter-days, whilst those that defined infection as positive culture plus clinical or CSF criteria also had a similar pooled VAI rate of 9.1/1000 catheter-days. Unsurprisingly, studies with a broader definition of infection, including positive culture or clinical features of meningo-ventriculitis, or abnormal CSF parameters, had a much higher rate of 17/1000 catheter-days. Strict definitions that insist upon positive CSF culture may miss some VAI when AIC are used as AIC reduce the chance of a positive culture [50]. Failure to process CSF in anaerobic media with prolonged incubation is another cause of negative cultures.

The effect of duration of ventricular catheterization on VAI rate is controversial [1]. Some institutions practice mandatory ventriculostomy revision after a certain duration [25], though mandatory revisions have not been shown to reduce VAI rate [18]. The evidence for increased duration causing an increased risk of VAI is conflicting with some studies suggesting an increasing risk with duration [3,11,13,25,51,52] longer than 5–7 days, with others showing no association [4,53-56]. In our analysis, we found decreasing VAI rate with increasing mean duration of ventricular catheterization when the studies were grouped according to duration less than 7 days, 7–10 days and greater than 10 days. The estimates were 19.6, 12.8 and 8 per 1000 catheter-days respectively. Whilst this is an unadjusted analysis that does not control for confounders, duration of treatment being a risk factor for VAI cannot be supported based on this data. Further, presence or suspicion of VAI will lead to removal and replacement of the ventricular catheter, whereas a non-infected catheter will tend to be left in situ as long as clinically indicated. Therefore, to some extent, it can be expected that the VAI rate will be higher amongst catheters with a shorter duration.

The main strengths of this meta-analysis are thorough literature search(including non-English articles) to identify all relevant published material, large number of included studies, large number of catheter-days of observation and infections and low likelihood of publication bias as detected by the funnel plot. The overall quality of studies, as assessed by the NOS, was high. Whilst efforts were made to locate all relevant published studies, unpublished data were not sought. It is plausible that this may have a significant impact on the pooled estimate of VAI rate. It is commonplace for institutions to audit their hospital-acquired infection rates. It is possible that such datasets exist in unpublished documents. This meta-analysis was also limited by reporting of VAI in formats that were not convertible to rate per 1000 catheter-days. Most of these studies expressed VAI as percentage of patients or ventriculostomies. Some causes of heterogeneity were explored in the subgroup analyses, however in this study, being a meta-analysis of cohort studies we were limited by the data that individual studies published.

Future research should focus on addressing the limitations of this meta-analysis, particularly on sourcing unpublished data from around the world, to assess the impact of this data on the results of our study. Data reporting in such a format that allows calculation of rate of events per person-time at risk should be encouraged as a standard format that allows comparisons across cohorts and pooling in meta-analyses. Participation in multicenter ventriculostomy registries should be encouraged as this will facilitate larger sample sizes, standardized data collection, exploration of heterogeneity, increased collaboration between researchers and individual patient data meta-analysis. A large, prospective observational or population study is required to confirm the results and explore the reasons for heterogeneity presented in this review. Further analyses of risk factors, as well as identification of preventative measures for VAI could be addressed in such a study.

## Conclusion

Ventriculostomies are common neurosurgical interventions frequently complicated by CSF infection. This meta-analysis has found that the VAI rate is 11.4/1000 catheter-days, or 12.1/1000 catheter-days with AIC removed. The rates across different age groups, study types, CSF culture regimes and common VAI definitions were similar, whilst being lower amongst studies with longer mean duration of ventriculostomy treatment and with AIC usage. The majority of positive cultures were gram positive bacteria, but 35% of positive cultures were gram negatives, the most common being *Acinetobacter* and *Pseudomonas* species, with subsequent implications for empirical antibiotic therapy. Future directions include more rigorous analysis of risk factors for VAI and confirmation of these data with large prospective, observational studies.

## References

[1] Beer R, Lackner P, Pfausler B, Schmutzhard E (2008). Nosocomial ventriculitis and meningitis in neurocritical care patients. J Neurol.

[2] Hoefnagel D, Dammers R, Ter Laak-Poort MP, Avezaat CJJ (2008). Risk factors for infections related to external ventricular drainage. Acta Neurochir.

[3] Wyler AR, Kelly WA (1972). Use of antibiotics with external ventriculostomies. J Neurosurg.

[4] Smith RW, Alksne JF (1976). Infections complicating the use of external ventriculostomy. J Neurosurg.

[5] Smigoc T, Rink N, Beovic B, Bosnjak R (2012). Risk factors for infections related to external ventricular drainage. Zdravniski Vestnik.

[6] Kitchen WJ, Singh N, Hulme S, Galea J, Patel HC, King AT (2011). External ventricular drain infection: improved technique can reduce infection rates. Br J Neurosurg.

[7] Mahe V, Kermarrec N, Ecoffey C (1995). Infections related to external ventricular drainage. Ann Francaises d Anesth Reanim.

[8] Camacho EF, Boszczowski I, Basso M, Jeng BCP, Freire MP, Guimaraes T, Teixeira MJ, Costa SF (2011). Infection rate and risk factors associated with infections related to external ventricular drain. Infection.

[9] Khalil BA, Sarsam Z, Buxton N (2005). External ventricular drains: is there a time limit in children?. Childs Nerv Syst.

[10] Dasic D, Hanna SJ, Bojanic S, Kerr RSC (2006). External ventricular drain infection: the effect of a strict protocol on infection rates and a review of the literature. Br J Neurosurg.

[11] Schultz M, Moore K, Foote AW (1993). Bacterial ventriculitis and duration of ventriculostomy catheter insertion. J Neurosci Nurs.

[12] Moon HJ, Kim SD, Lee JB, Lim DJ, Park JY (2007). Clinical analysis of external ventricular drainage related ventriculitis. J Korean Neurosurg Soc.

[13] Lyke KE, Obasanjo OO, Williams MA, O’Brien M, Chotani R, Perl TM (2001). Ventriculitis complicating use of intraventricular catheters in adult neurosurgical patients. Clin Infect Dis.

[14] Williams TA, Leslie GD, Dobb GJ, Roberts B, van Heerden PV (2011). Decrease in proven ventriculitis by reducing the frequency of cerebrospinal fluid sampling from extraventricular drains. J Neurosurg.

[15] Rafiq MFA, Ahmed N, Ali S (2011). Effect of tunnel length on infection rate in patients with external ventricular drain. J Ayub Med Coll Abbottabad.

[16] Lundberg F, Wady L, Soderstrom S, Siesjo P, Larm O, Ljungh A (2000). External ventricular drainage catheters: effect of surface heparinization on bacterial colonization and infection. Acta Neurochir.

[17] Hader WJ, Steinbok P (2000). The value of routine cultures of the cerebrospinal fluid in patients with external ventricular drains. Neurosurgery.

[18] Lo CH, Spelman D, Bailey M, Cooper DJ, Rosenfeld JV, Brecknell JE (2007). External ventricular drain infections are independent of drain duration: an argument against elective revision. J Neurosurg.

[19] McLaughlin N, St-Antoine P, Bojanowski MW (2012). Impact of antibiotic-impregnated catheters on the timing of cerebrospinal fluid infections in non-traumatic subarachnoid hemorrhage. Acta Neurochir (Wien).

[20] Lemcke J, Depner F, Meier U (2012). The impact of silver nanoparticle-coated and antibiotic-impregnated external ventricular drainage catheters on the risk of infections: a clinical comparison of 95 patients. Acta Neurochir Suppl.

[21] Leverstein-van Hall MA, Hopmans TEM, van der Sprenkel JWB, Blok HEM, van der Mark WAMA, Hanlo PW, Bonten MJM (2010). A bundle approach to reduce the incidence of external ventricular and lumbar drain-related infections. J Neurosurg.

[22] Scheithauer S, Burgel U, Bickenbach J, Hafner H, Haase G, Waitschies B, Reinges MHT, Lemmen SW (2010). External ventricular and lumbar drainage-associated meningoventriculitis: prospective analysis of time-dependent infection rates and risk factor analysis. Infection.

[23] Scheithauer S, Burgel U, Ryang YM, Haase G, Schiefer J, Koch S, Hafner H, Lemmen S (2009). Prospective surveillance of drain associated meningitis/ventriculitis in a neurosurgery and neurological intensive care unit. J Neurol Neurosurg Psychiatry.

[24] Chi H, Chang K-Y, Chang H-C, Chiu N-C, Huang F-Y (2009). Infections associated with indwelling ventriculostomy catheters in a teaching hospital. Int J Infect Dis.

[25] Holloway KL, Barnes T, Choi S, Bullock R, Marshall LF, Eisenberg HM, Jane JA, Ward JD, Young HF, Marmarou A (1996). Ventriculostomy infections: the effect of monitoring duration and catheter exchange in 584 patients. J Neurosurg.

[26] Rivero-Garvia M, Marquez-Rivas J, Jimenez-Mejias ME, Neth O, Rueda-Torres AB (2011). Reduction in external ventricular drain infection rate. Impact of a minimal handling protocol and antibiotic-impregnated catheters.[Erratum appears in Acta Neurochir (Wien). 2011 May;153(5):1157]. Acta Neurochirurgica.

[27] Lwin S, Low SW, Choy DKS, Yeo TT, Chou N (2012). External ventricular drain infections: successful implementation of strategies to reduce infection rate. Singapore Med J.

[28] Fichtner J, Guresir E, Seifert V, Raabe A (2010). Efficacy of silver-bearing external ventricular drainage catheters: a retrospective analysis. J Neurosurg.

[29] Park PMD, Garton HJLMDMHS, Kocan MJRNMSN, Thompson BGMD (2004). Risk of infection with prolonged ventricular catheterization. Neurosurgery.

[30] Roitberg BZ, Khan N, Alp MS, Hersonskey T, Charbel FT, Ausman JI (2001). Bedside external ventricular drain placement for the treatment of acute hydrocephalus. Br J Neurosurg.

[31] Alleyne CH, Hassan M, Zabramski JM (2000). The efficacy and cost of prophylactic and perioprocedural antibiotics in patients with external ventricular drains. Neurosurgery.

[32] Schodel P, Proescholdt M, Brawanski A, Bele S, Schebesch K-M (2012). Ventriculostomy for acute hydrocephalus in critically ill patients on the ICU–outcome analysis of two different procedures. Br J Neurosurg.

[33] Sloffer CA, Augspurger L, Wagenbach A, Lanzino G (2005). Antimicrobial-impregnated external ventricular catheters: does the very low infection rate observed in clinical trials apply to daily clinical practice?. Neurosurgery.

[34] Kim J-H, Desai NS, Ricci J, Stieg PE, Rosengart AJ, Hartl R, Fraser JF (2012). Factors contributing to ventriculostomy infection. World Neurosurg.

[35] Bota DP, Lefranc F, Vilallobos HR, Brimioulle S, Vincent JL (2005). Ventriculostomy-related infections in critically ill patients: a 6-year experience. J Neurosurg.

[36] Arabi Y, Memish ZA, Balkhy HH, Francis C, Ferayan A, Al Shimemeri A, Almuneef MA (2005). Ventriculostomy-associated infections: incidence and risk factors. Am J Infect Control.

[37] Moher D, Liberati A, Tetzlaff J, Altman DG (2009). Preferred reporting items for systematic reviews and meta-analyses: the PRISMA statement. Ann Intern Med.

[38] Lozier APMD, Sciacca RRESD, Romagnoli MFMD, Connolly ESJMD. Ventriculostomy-related infections: a critical review of the literature. Neurosurg Surg Hum Cerebrum. 2008;62(Supplement(2)):SHC-688–SHC-700. 10.1227/01.neu.0000316273.35833.7c 18596436

[39] Ratilal B, Costa J, Sampaio C (2008). Antibiotic prophylaxis for surgical introduction of intracranial ventricular shunts: a systematic review. J Neurosurg Pediatr.

[40] Thomas R, Lee S, Patole S, Rao S (2012). Antibiotic-impregnated catheters for the prevention of CSF shunt infections: a systematic review and meta-analysis. Br J Neurosurg.

[41] Sonabend AM, Korenfeld Y, Crisman C, Badjatia N, Mayer SA, Connolly ES (2011). Prevention of ventriculostomy-related infections with prophylactic antibiotics and antibiotic-coated external ventricular drains: a systematic review. Neurosurgery.

[42] The Newcastle-Ottawa Scale (NOS) for assessing the qualities of nonrandomised studies in meta-analyses [http://www.ohri.ca/programs/clinical_epidemiology/oxford.asp]

[43] Neyeloff JL, Fuchs SC, Moreira LB. Meta-analyses and Forest plots using a microsoft excel spreadsheet: step-by-step guide focusing on descriptive data analysis. BMC Res Notes. 2012;5:52. 10.1186/1756-0500-5-52 PMC3296675 22264277

[44] DerSimonian R, Laird N (1986). Meta-analysis in clinical trials. Control Clin Trials.

[45] Higgins JP, Thompson SG, Deeks JJ, Altman DG (2003). Measuring inconsistency in meta-analyses. BMJ (Clin Res ed).

[46] Egger M, Davey Smith G, Schneider M, Minder C (1997). Bias in meta-analysis detected by a simple, graphical test. BMJ (Clin Res ed).

[47] Garner J, Jarvis W, Emori T, Horan T, Hughes J, Olmsted R (1996). CDC Definitions for nosocomial infections. APIC Infection control and applied epidemiology: Principles and practice.

[48] Eymann R, Chehab S, Strowitzki M, Steudel WI, Kiefer M (2008). Clinical and economic consequences of antibiotic-impregnated cerebrospinal fluid shunt catheters. J Neurosurg Pediatr.

[49] Korinek AM, Reina M, Boch AL, Rivera AO, De Bels D, Puybasset L (2005). Prevention of external ventricular drain–related ventriculitis. Acta Neurochir.

[50] Stevens EA, Palavecino E, Sherertz RJ, Shihabi Z, Couture DE (2010). Effects of antibiotic-impregnated external ventricular drains on bacterial culture results: an in vitro analysis. J Neurosurg.

[51] Mayhall CG, Archer NH, Lamb VA, Spadora AC, Baggett JW, Ward JD, Narayan RK (1984). Ventriculostomy-related infections. A prospective epidemiologic study. New England Journal of Medicine.

[52] Paramore CG, Turner DA (1994). Relative risks of ventriculostomy infection and morbidity. Acta Neurochir.

[53] Ohrstrom JK, Skou JK, Ejlertsen T, Kosteljanetz M (1989). Infected ventriculostomy: bacteriology and treatment. Acta Neurochir (Wien).

[54] Stenager E, Gerner-Smidt P, Kock-Jensen C (1986). Ventriculostomy-related infections–an epidemiological study. Acta Neurochir.

[55] Sundbarg G, Nordstrom CH, Soderstrom S (1988). Complications due to prolonged ventricular fluid pressure recording. Br J Neurosurg.

[56] Winfield JA, Rosenthal P, Kanter RK, Casella G (1993). Duration of intracranial pressure monitoring does not predict daily risk of infectious complications. Neurosurgery.

